# From Maxwell's Equations to Polarimetric SAR Images: A Simulation Approach

**DOI:** 10.3390/s8117380

**Published:** 2008-11-19

**Authors:** Sidnei J. S. Sant'Anna, J. C. da S. Lacava, David Fernandes

**Affiliations:** 1 Divisão de Processamento de Imagem, Instituto Nacional de Pesquisas Espaciais, Avenida dos Astronautas, 1758, CEP 12227-010, São José dos Campos - SP, Brazil; 2 Laboratório de Antenas e Propagação, Instituto Tecnológico de Aeronáutica, Praça Mal. Eduardo Gomes, 50, CEP 12228-900, São José dos Campos - SP, Brazil;

**Keywords:** Synthetic Aperture Radar, Polarimetric SAR image simulation, electromagnetic scattering model, planar multilayer structure, SAR data analysis

## Abstract

A new electromagnetic approach for the simulation of polarimetric SAR images is proposed. It starts from Maxwell's equations, employs the spectral domain full-wave technique, the moment method, and the stationary phase method to compute the far electromagnetic fields scattered by multilayer structures. A multilayer structure is located at each selected position of a regular rectangular grid of coordinates, which defines the scene area under imaging. The grid is determined taking into account the elementary scatter size and SAR operational parameters, such as spatial resolution, pixel spacing, look angle and platform altitude. A two-dimensional separable “sinc” function to represent the SAR spread point function is also considered. Multifrequency sets of single-look polarimetric SAR images are generated, in L-, C- and X-bands and the images are evaluated using several measurements commonly employed in SAR data analysis. The evaluation shows that the proposed simulation process is working properly, since the obtained results are in accordance with those presented in the literature. Therefore, this new approach becomes suitable for carrying out theoretical and practical studies using polarimetric SAR images.

## 1. Introduction

Retrieval of targets' biophysical and geophysical parameters is one of the main goals of microwave remote sensing, having been the subject of numerous studies. Understanding the electromagnetic reflective properties of targets is a key to the correct interpretation of microwave imaging data. In this sense an image simulator might become a powerful tool for remote sensing researchers, since the use of simulated images may improve considerably the knowledge on several synthetic aperture radar (SAR) applications.

The applicability of synthesized images ranges from theoretical considerations to practical problems. For instance, from SAR simulated images it is possible to develop dedicated algorithms for filtering, segmenting or classifying images. Simulated images can also be used in remote sensing inversion techniques, in the identification of the scattering mechanisms intrinsic to a set of pixels, and in sensor calibration procedures, among others.

There are several ways to synthesize an image; for instance, in [1-3] a statistical technique is used to generate amplitude SAR data. The employed distributions are derived from multiplicative models and are associated with the homogeneity degree of each target [4]. However, to perform the simulation process as realistic as possible, the polarimetric SAR image simulation problem is addressed in this work from the point of view of electromagnetic modeling. The employed simulation approach is based on the computation of the far electric field scattered by a multilayer structure excited by a plane wave, using the moment method technique [5].

According to [6], the next challenging era of satellite programmes are the Cartwheel satellites, consisting of a transmitter and several small receivers for a specific purpose. This technique is completely based on the bistatic behavior of radar waves for different targets. Therefore, there is currently a need to perform such bistatic radar measurements for various kinds of targets and to develop a less complex model for the retrieval of the target characteristics. There are currently in the literature few works that have used the polarimetric analysis of bistatic radar data to retrieve target parameters, such as [7]. Although the SAR image simulation process proposed here can be used in either monostatic or bistatic SAR framework without any additional complexity, this paper assumes the monostatic framework to simulate polarimetric SAR images.

The paper is organized as follows. A general theory behind the electromagnetic model is outlined in Section 2. In this section the moment method is also developed and applied to a particular structure. The generation of polarimetric SAR images is detailed in Section 3, where SAR images for a simple multilayer structure are simulated. In Section 4, the simulated polarimetric images are evaluated based on intrinsic properties of amplitude and polarimetric SAR data; results of different classification approaches are also analyzed. Finally, the conclusions are drawn in Section 5.

## 2. Electromagnetic Model

**Figure 1.**
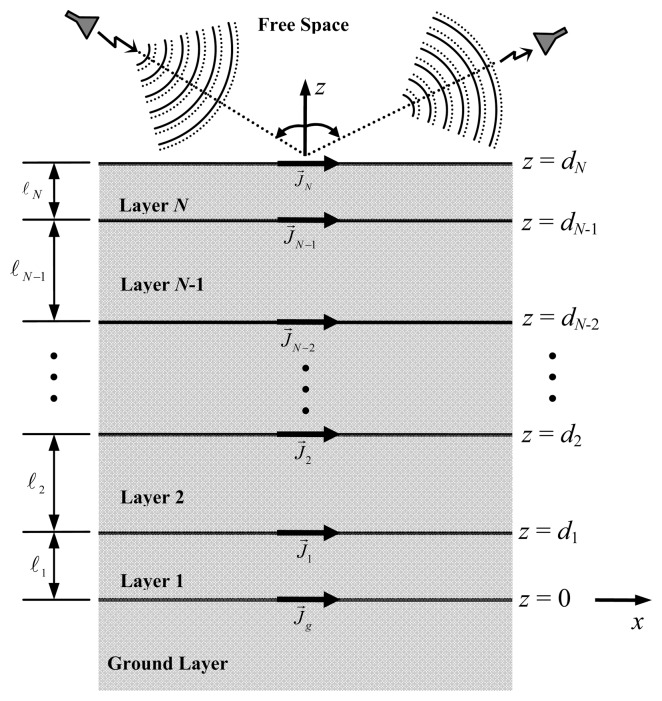
Geometry of the planar structure with *N* + 2 layers (lateral view).

The electromagnetic model is based on the determination of the electromagnetic fields scattered by a multilayer planar structure that is excited by plane waves. The structure under analysis is composed of *N*+2 isotropic, linear and homogenous layers stacked up in *z* direction. The layers are assumed to be unbounded along the *x* and *y* directions. The lower layer, having complex permittivity *ε_g_* and complex permeability *μ_g_*, is denoted as ground layer and occupies the negative-*z* region. The next *N* layers are characterized by thickness *ℓ_n_*, complex permittivity *ε_n_* and complex permeability *μ_n_*, where 1 ≤ *n* ≤ *N*. The planar interface *z* = *d_N_* separates the *N*-th layer from free space (the upper layer). Metallic patches, which behave as scattering elements, are printed at arbitrary positions on each one of the *N*+1 interfaces of the structure. The development is based on a global right-handed rectangular coordinate system located on the top of the ground layer (interface *z* = 0) and lying on the *xy*-plane. The geometry of the planar multilayer structure is depicted in Figure 1.

### 2.1. Electromagnetic Fields in the Structure

The electromagnetic fields in a multilayer structure are determined through the methodological approach described in [8]. According to this methodology, which employs the spectral domain full-wave technique, the structure is treated as a boundary value problem, where the induced electric surface current densities on the metallic patches are the virtual sources of the scattered fields. Since the layers of the structure are free of sources, the Maxwell's equations for the *n*-th layer, assuming time dependence of the form *e^jωt^*, are

(1) $$\nabla \times {\text{E}}_{n}(x,y,z)=-i\omega {\text{B}}_{n}(x,y,z),$$

(2) $$\nabla \times {\text{H}}_{n}(x,y,z)=i\omega {\text{D}}_{n}(x,y,z),$$

(3) $$\nabla \cdot {\text{D}}_{n}(x,y,z)=0,$$

(4) $$\nabla \cdot {\text{B}}_{n}(x,y,z)=0,$$

where, for free space and the ground layer the index *n* is equal to 0 and *g* respectively, *ω* is the angular frequency and the vectors ***E****_n_*(*x*, *y*, *z*), ***H****_n_*(*x*, *y*, *z*), ***D****_n_*(*x*, *y*, *z*), and ***B****_n_*(*x*, *y*, *z*) denote the complex electric field, magnetic field, electric flux density and magnetic flux density, respectively (bold face letters represent vectors).

Using the following constitutive relations for each of these media

(5) $${\text{D}}_{n}(x,y,z)=\varepsilon_{n}\;{\text{E}}_{n}(x,y,z),$$

(6) $${\text{B}}_{n}(x,y,z)=\mu_{n}\;{\text{H}}_{n}(x,y,z),$$

the wave equations for the *n*-th layer is written as

(7) $$\nabla^{2}{\text{E}}_{n}(x,y,z)+k_{n}^{2}\;{\text{E}}_{n}(x,y,z)=0,$$

(8) $$\nabla^{2}{\text{H}}_{n}(x,y,z)+k_{n}^{2}\;{\text{H}}_{n}(x,y,z)=0,$$

where $k_{n}^{2}=\omega^{2}\;\mu_{n}\varepsilon_{n}$ gives the wave number in the *n*-th layer. The wave equations can be solved in the spectral domain using the double Fourier transform. In this paper, the Fourier transform pair is defined as

(9) $$F(k_{x},k_{y},z)=\iint_{-\infty }^{+\infty }F(x,y,z)e^{i(k_{x}x+k_{y}y)}\text{dxdy},$$

(10) $$F(x,y,z)=\frac{1}{4\pi^{2}}\iint_{-\infty }^{+\infty }F(k_{x},k_{y},z)e^{-i(k_{x}x+k_{y}y)}dk_{x}dk_{y},$$

where the *F*(*x*, *y*, *z*) function represents the fields ***E****_n_*(*x*, *y*, *z*) or ***H****_n_*(*x*, *y*, *z*). Application of the double Fourier transform to (7) and (8) yields a differential equation system whose general solution, in terms of the fields components, is given by

(11) $${ℰ}_{n\vartheta }(k_{x},k_{y},z)=e_{n\vartheta }(k_{x},k_{y})e^{i\gamma_{n}z},$$

(12) $${ℋ}_{n\vartheta }(k_{x},k_{y},z)=h_{n\vartheta }(k_{x,}k_{y})e^{i\gamma_{n}z},$$

with

(13) γn=(−1)τkn2−(kx2+ky2)Im(γn)≤0,

where 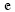
*_nϑ_* (*k_x_*,*k_y_*) are 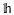
*_nϑ_* (*k_x_*,*k_y_*) the amplitudes of the transformed field components, *k_x_* and *k_y_* are the spectral variables, *γ_n_* is the propagation constant in the *n*-th layer, *ϑ* = *x*, *y* or *z*, and Im( ) means the imaginary-part function. The *τ* variable, which defines the wave propagation direction, can assume values 1 or 2. Only the former value, representing propagation in the positive-*z* direction, occurs in the upper layer (free space). For the ground layer, on the other hand, *τ* equals 2, i.e., a wave propagating in the negative-*z* direction. For the confined layers, however, both values of *τ* will occur.

Interesting relations among the amplitudes of the transformed fields are derived by introducing the inverse Fourier transform of (11) and (12) in the Maxwell's curl equations, such that the amplitudes of the transversal components (*x* and *y* directions) are written as functions of the amplitude of the longitudinal ones (*z* direction). By enforcing the boundary conditions for the electromagnetic fields at each interface a set of 4*N*+4 equations with an equal number of unknowns is obtained. The analytical solution of this system leads to the spectral Green's functions. These functions, jointly with the transformed superficial density currents, allow the determination of the transformed fields at any point of the multilayer structure. The transformed electromagnetic field components are expressed by

(14) $${ℰ}_{n\vartheta }(k_{x},k_{y},d_{v})=\sum_{v}G_{\vartheta vx}^{\left(n\right)}(k_{x},k_{y},d_{v})\;j_{vx}(k_{x},k_{y})+\sum_{v}G_{\vartheta vy}^{\left(n\right)}(k_{x},k_{y},d_{v})\;j_{vy}(k_{x},k_{y}),$$

(15) $$H_{n\vartheta }(k_{x},k_{y},d_{v})=\sum_{v}Q_{\vartheta vx}^{\left(n\right)}(k_{x},k_{y},d_{v})\;j_{vx}(k_{x},k_{y})+\sum_{v}Q_{\vartheta vy}^{\left(n\right)}(k_{x},k_{y},d_{v})\;j_{vy}(k_{x},k_{y}),$$

where $G_{\vartheta v\varsigma }^{\left(n\right)}(k_{x},k_{y},dv)$ and $Q_{\vartheta v\varsigma }^{\left(n\right)}(k_{x},k_{y},dv)$ represent, respectively, the electrical and magnetic spectral Green's functions in the *n*-th layer, which relate the *ς* (*ς* = *x* or *y*) components of the electric and magnetic fields to the transformed superficial density current *j_vς_*(*k_x_*, *k_y_*) located at the interface *d_v_*, with *v ∈* {*g*, 1, 2, …, *N*}. Note that *d_g_* = 0.

### 2.2. Moment Method - MoM

Once the Green's functions are derived, the next step is to set up integral equations constrained to the required boundary conditions. The integral equation is a statement of the boundary condition requiring that the total electric field tangential to the each of the perfectly conducting surfaces is zero [9]. That is,

(16) $$\hat{z}\times [{\text{E}}^{i}(x,y,d_{v})+{\text{E}}^{r}(x,y,d_{v})]=-\hat{z}\times {\text{E}}^{s}(x,y,d_{v}),\;\mathrm{on}\;S_{v}$$

where *S_v_* are the conducting surfaces, ***E****^s^* (*x*, *y*, *d_v_*) denotes the scattered field excited by the current on *S_v_*, ***E****^i^*(*x*, *y*, *d_v_*) stands for the incident electric field and ***E****^r^*(*x*, *y*, *d_v_*) identifies the field that is reflected by the multilayer structure in the absence of patches. The ***E****^i^*(*x*, *y*, *d_v_*) and ***E****r*(*x*, *y*, *d_v_*) fields define the excitation mechanism of the structure, which in this analysis is due to an elliptically polarized plane wave at an arbitrary incidence angle. The currents induced on the conducting surfaces by these fields are unknown. To solve the electric field integral Equation (16), with the unknown surface currents, the moment method is applied. This method is one the most popular numerical techniques used to analyze the radiation and scattering from complex structures. In the MoM, first the surface current is linearly expanded in a set of basis functions with unknown coefficients

(17) $${\text{j}}_{v}(k_{x},k_{y})=\hat{x}\sum_{m=1}^{M_{\text{Lv}}}\sum_{n=1}^{N_{\text{Lv}}}I_{\text{mvnv}}^{x}\;j_{\text{mv}}(k_{x},k_{y})+\hat{y}\sum_{m=1}^{M_{\text{Lv}}}\sum_{n=1}^{N_{\text{Lv}}}I_{\text{mvnv}}^{y}\;j_{\text{nv}}(k_{x},k_{y}),$$

where *M_L_* and *N_L_* control the expansion modes in *x* and *y* directions on each interface layer, respectively, $I_{\text{mvnv}}^{\varsigma }$ are the complex coefficients in the *ς* direction (*ς* = *x* or *y*) that need to be determined, and *j_m__v_*(*k_x_*, *k_y_*) and *j_n__v_*(*k_x_*, *k_y_*) are the Fourier transform of the surface density current components, which are defined only over the conducting surface. Applying the Galerkin technique (whereby the test functions are chosen to be identical to the basis functions) the integral equation is reduced to a system of simultaneous linear equations, which can be compactly written in matrix form as [*V*] = [*Z*][*I*]. In this notation [*V*], [*Z*] and [*I*] denote, respectively, the excitation matrix, the impedance matrix and the coefficient matrix. For example, the integral equation referring to the scattered fields at the interface *z* = *d_n_* can be written as:

(18) $$\begin{array}{c} 4\pi^{2}\iint_{S_{n}}E_{0x}^{s}\;(x,y,d_{n})\;j_{\text{pn}}^{*}(x,y)\;\text{dx}\;\text{dy}=\sum_{v=0}^{N}\sum_{m=1}^{M_{\text{Lv}}}\sum_{n=1}^{N_{\text{Lv}}}I_{\text{mvnv}}^{x}\int_{-\infty }^{+\infty }\int_{-\infty }^{+\infty }G_{\text{xvx}}^{\left(0\right)}\;j_{\text{mv}}\;j_{\text{pn}}^{*}\;dk_{x}\;dk_{y}\\ +\sum_{v=0}^{N}\sum_{m=1}^{M_{\text{Lv}}}\sum_{n=1}^{N_{\text{Lv}}}I_{\text{mvnv}}^{y}\int_{-\infty }^{+\infty }\int_{-\infty }^{+\infty }G_{\text{xvy}}^{\left(0\right)}\;j_{\text{nv}}\;j_{\text{pn}}^{*}\;dk_{x}\;dk_{y}, \end{array}$$

(19) $$\begin{array}{c} 4\pi^{2}\iint_{S_{n}}E_{0y}^{s}\;(x,y,d_{n})\;j_{\text{qn}}^{*}\;(x,y)\;\text{dx}\;\text{dy}=\sum_{v=0}^{N}\sum_{m=1}^{M_{\text{Lv}}}\sum_{n=1}^{N_{\text{Lv}}}I_{\text{mvnv}}^{x}\int_{-\infty }^{+\infty }\int_{-\infty }^{+\infty }G_{\text{yvx}}^{\left(0\right)}\;j_{\text{mv}}\;j_{\text{qn}}^{*}\;dk_{x}\;dk_{y}\\ +\sum_{v=0}^{N}\sum_{m=1}^{M_{\text{Lv}}}\sum_{n=1}^{N_{\text{Lv}}}I_{\text{mvnv}}^{y}\int_{-\infty }^{+\infty }\int_{-\infty }^{+\infty }G_{\text{yvy}}^{\left(0\right)}\;j_{\text{nv}}\;j_{\text{qn}}^{*}\;dk_{x}\;dk_{y}, \end{array}$$

where the left sides of (18) and (19) define the [*V*] matrix and the double integrals are related to the [*Z*] matrix.

The double integrations in Equation (18) and (19) must be performed numerically, usually in a very inefficient and time-consuming way. In order to improve the computation efficiency some mathematical simplifications are employed. These simplifications include the evaluation of the even and the odd properties of the Green's functions, the change of the coordinate system (rectangular to polar) and the asymptotic extraction technique.

The far electromagnetic fields scattered by the multilayer structure are computed based on asymptotic expressions, which are derived from the stationary phase method [10]. The electric far field, using the stationary phase method, is given by

(20) $${\text{E}}_{0}(r,\theta ,\phi )≅-\frac{ik_{0}}{2\pi }\frac{e^{-ik_{0}r}}{r}\cot\theta \left\{\hat{\theta }e_{0z}(k_{\text{xe}},k_{\text{ye}})-\hat{\phi }\eta_{0}h_{0z}(k_{\text{xe}},k_{\text{ye}})\right\},$$

in a spherical coordinate *r* system, where the intrinsic impedance of free space is represented by *η*_0_, *k_xe_* = *k*_0_ sin*θ*cos*ϕ* and *k_ye_* = *k*_0_ sin*θ*sin*ϕ* are the stationary phase points, *k*_0_ is the wave number of the excitation wave and *r* characterizes the distance between the receiving antenna and the target. Notice that from the knowledge of the electric far field it is possible to calculate the scattering matrix elements.

### 2.3. Four-Layer Structure

**Figure 2.**
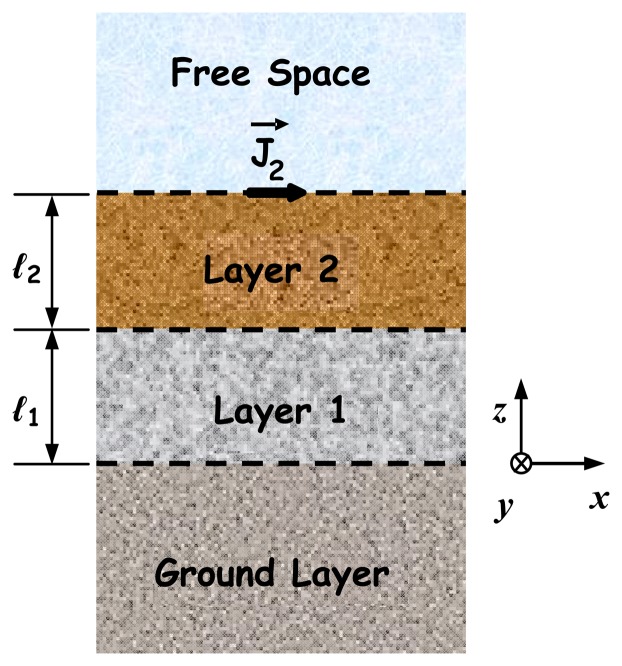
Geometry of a planar structure with four layers (lateral view).

The approach described above is now applied to a structure, consisting of four layers (*N* = 2), as illustrated in Figure 2. A flat electric dipole of infinitesimal thickness is selected to represent the metallic patch. The dipole, printed on the interface *z* = *d*_2_ (*d*_2_ =*ℓ*_1_ + *ℓ*_2_) and oriented along the *x* direction, has dimensions 2*a* and 2*b* (*a* ≫ *b*) in the *x* and the *y* directions respectively. For this structure, the amplitudes of the transformed far field components (in the *z* direction) in free space are given by

(21) $$e_{0z}(k_{x},k_{y})=\frac{4\omega^{2}\Omega_{0}^{e}}{\Delta_{e}}\left\{k_{x}\;j_{2x}(k_{\text{xe}},k_{\text{ye}})+k_{y}\;j_{2y}(k_{\text{xe}},k_{\text{ye}})\right\},$$

(22) $$h_{0z}(k_{x},k_{y})=\frac{4\omega^{2}\Omega_{0}^{h}}{\Delta_{m}}\left\{k_{y}\;j_{2x}(k_{\text{xe}},k_{\text{ye}})-k_{x}\;j_{2y}(k_{\text{xe}},k_{\text{ye}})\right\},$$

(23) $$\Omega_{0}^{e}=\gamma_{2}\{\varepsilon_{1}\gamma_{2}(\varepsilon_{1}\gamma_{g}\sin\alpha_{1}-i\varepsilon_{g}\gamma_{1}\cos\alpha_{1})\sin\alpha -\varepsilon_{2}\gamma_{1}(\varepsilon_{1}\gamma_{g}\cos\alpha_{1}+i\varepsilon_{g}\gamma_{1}\sin\alpha_{1})\cos\alpha \},$$

(24) $$\Omega_{0}^{h}=\omega \mu_{2}\{\mu_{1}\gamma_{2}(\mu_{g}\gamma_{1}\cos\alpha_{1}+i\mu_{1}\gamma_{g}\sin\alpha_{1})\cos\alpha -\mu_{2}\gamma_{1}(\mu_{g}\gamma_{1}\sin\alpha_{1}-i\mu_{1}\gamma_{g}\cos\alpha_{1})\sin\alpha \},$$

(25) $$\Delta_{e}=-4\omega^{3}e^{-i\alpha 0}\{\varepsilon_{1}\gamma_{2}(\varepsilon_{2}\gamma_{0}\cos\alpha +i\varepsilon_{0}\gamma_{2}\sin\alpha )(\varepsilon_{g}\gamma_{1}\cos\alpha_{1}+i\varepsilon_{1}\gamma_{g}\sin\alpha_{1})+\varepsilon_{2}\gamma_{1}(\varepsilon_{0}\gamma_{2}\cos\alpha +i\varepsilon_{2}\gamma_{0}\sin\alpha )(\varepsilon_{1}\gamma_{g}\cos\alpha_{1}+i\varepsilon_{g}\gamma_{1}\sin\alpha_{1})\},$$

(26) $$\Delta_{m}=4\omega^{3}e^{-i\alpha 0}\{\mu_{1}\gamma_{2}(\mu_{2}\gamma_{0}\cos\alpha +i\mu_{0}\gamma_{2}\sin\alpha )(\mu_{g}\gamma_{1}\cos\alpha_{1}+i\mu_{1}\gamma_{g}\sin\alpha_{1})+\mu_{2}\gamma_{1}(\mu_{0}\gamma_{2}\cos\alpha +i\mu_{2}\gamma_{0}\sin\alpha )(\mu_{1}\gamma_{g}\cos\alpha_{1}+i\mu_{g}\gamma_{1}\sin\alpha_{1})\},$$

where *α*_0_ = *γ*_0_*d*_2_, *α*_1_ = *γ*_1_*d*_1_, and *α* = *γ*_2_ (*d*_2_−*d*_1_). Notice that only the amplitudes 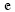
_0_*_z_* (*k_x_*, *k_y_*) and 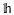
_0_*_z_* (*k_x_*, *k_y_*) are necessary to compute the electric far field, as shown in (20). In this particular case, the surface current along the *y* direction is neglected since the dipole width is considered to be very thin. Thus only the $\left[Z_{p2m2}^{x2x2}\right]$ matrix, represented by [*Z_pm_*], involving the Green's function $G_{x2x}^{\left(0\right)}$, needs to be evaluated. After the aforementioned mathematical simplifications, the [*Z_pm_*] matrix becomes

(27) $$\left[Z_{\text{pm}}\right]=\frac{1}{\pi^{2}}\left\{\int_{\beta =0}^{+\infty }\int_{\alpha =0}^{\pi /2}[A_{1}{\left(\cos\alpha \right)}^{2}+A_{2}{\left(\sin\alpha \right)}^{2}]{\text{ℝ}}_{\text{pm}}\;d\alpha \;d\beta +\int_{\beta =0}^{+\infty }\int_{\alpha =0}^{\pi /2}\frac{i\;A_{3}}{\omega }{\left(\cos\alpha \right)}^{2}{\text{ℝ}}_{\text{pm}}\;d\alpha \;d\beta \right\},$$

with the factors *A*_1_, *A*_2_, *A*_3_ and *ℝ_pm_* given, respectively, by

(28) $$A_{1}=\frac{4\omega^{2}\;\beta \gamma_{0}\Omega_{0}^{e}}{\Delta_{e}}-\frac{i\;A_{3}}{\omega },$$

(29) $$A_{2}=\frac{4\omega^{3}\;\beta \mu_{0}\Omega_{0}^{h}}{\Delta_{m}},$$

(30) $$A_{3}=\frac{\beta^{2}}{\varepsilon_{0}+\varepsilon_{2}},$$

(31) $${\text{ℝ}}_{\text{pm}}=\pi^{2}b^{2}J_{0}^{2}(bk_{y})\sin c^{4}(k_{x}\Delta x/2)\cos[k_{x}(x_{m}-x_{p})].$$

Equation (31) is obtained from the modeling of the surface current density as the summation of piecewise-linear subdomain basis functions (rooftop functions) taking into account the edge condition. In this equation the sinc(.) is defined as sinc(*α*) = sin(*α*)/*α*, *J*_0_(.) stands for the zero-order Bessel function of the first kind and Δ*x* = 2*a*/(*M*+1).

**Figure 3.**
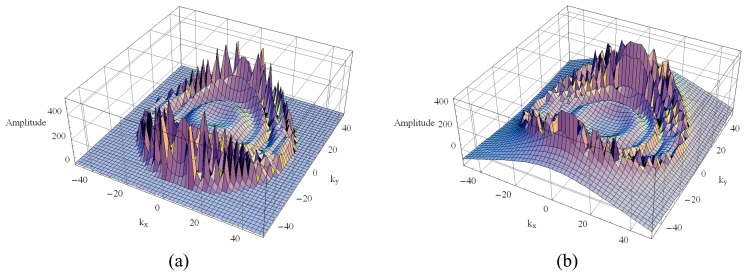
The 3-D Green's function at 1.25 GHz: (a) real part and (b) imaginary part.

**Figure 4.**
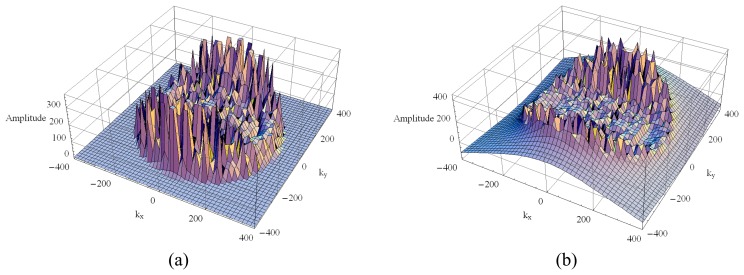
The 3-D Green's function at 9.6 GHz: (a) real part and (b) imaginary part.

From (27) to (31) it is noted that the first double integral of [*Z_pm_*] is dependent on the operating frequency, whereas the second one is not. These are the well-known Sommerfeld integrals, which exhibit singularities in the form of branch points and poles; as such, their computation requires careful attention. The poles (generally complex) correspond to surface and leaky waves that can be excited in the layers. According to [11] the number of poles and their locations depends on the thickness of the layers, their relative electric permittivity and the wave number. For a multilayer structure and depending on the thickness of the layers, the Green's functions might present hundreds of poles, making their integration a formidable task. As an example, Figures 3 and 4 illustrate the real and the imaginary parts of the Green's function of a four-layer structure for two different operating frequencies: 1.25 GHz and 9.6 GHz, respectively. The electric parameters that characterize this structure are: *ℓ*_1_ =*ℓ*_2_ = 263.82 mm, *ε_r_*_1_ = *ε_r_*_2_ = 2.33, tan*δ*_1_ = tan*δ*_2_ = 1.2×10^-4^, *ε_rg_* = 5.0 and tan*δ_g_* = 2.0×10^-1^. Notice that the layers 1 and 2 have the same electric characteristics, and the variations of the real and the imaginary parts of the Green's function become more numerous and more abrupt as the frequency increases. These graphics illustrate that neither the analytical nor the numerical treatment of this kind of function is an easy task.

**Figure 5.**
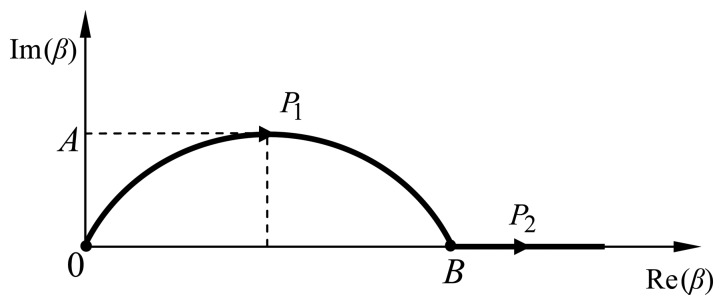
Parabolic deformed integration path.

The first double integral of (27) could be computed by a singularity extraction method, however this technique requires the calculation of residues and Cauchy principal values at the singularity points. A major problem in computing the poles' contribution is finding the accurate location of all poles in a region. Since the number of poles and their locations are not known beforehand [9], the use of a deformed path to compute the integrations seems to be an efficient way to avoid this problem. Therefore, in this work a parabolic path was chosen to compute the integrations over the interval [0, *B*] and then proceeding along the Re(*β*)-axis from *B* to ∞, assuming there are no singularities in the latter interval. The deformed contour *P*, which is defined by *P*_1_ and *P*_2_ paths, is depicted in Figure 5, where *P*_1_ represents the parabolic path and *P*_2_ the path along the real axis. The advantage of contour *P* is avoiding numerical integration near the poles. Further, no knowledge of the number of poles and their locations is required. Generally, the values of parameters *A* and *B* are, respectively, about 0.1*k*_0_ and 1.1*k*_0_*ε_rm_*, where *ε_rm_* = (max{*ε_r_*_1_, *ε_r_*_2_})^1/2^.

According to [12] the scattering of a flat electric dipole printed on the interface *z* = *d*_2_ of a four-layer structure can be accurately characterized by rooftop subdomain basis functions with twenty expansion modes and taking into account the edge condition. After that, the integrations are computed using the 96-point Gauss-Legendre quadrature rule, by truncating the upper limit of the *β* variable at 100,000 for the frequency-independent integral and at 50,000 for the frequency-dependent one, and by applying an additional subdivision technique, consisting of 10 subintervals, to compute the integral of the *β* variable. In this work, such MoM setup was used to compute the scattering of the printed dipole for simulation process purposes.

## 3. Polarimetric SAR Image Simulation

To exemplify the process of polarimetric SAR image simulation, the four-layer structure presented in Section 2.3 is considered. The parameters that can be varied in this structure are: the thickness of each confined layer, the dielectric characteristics of each layer (except for free space), and the size, orientation and location of the dipoles. Meanwhile, to define an image region having similar electromagnetic characteristic in its pixels, only dipole orientation is varied whereas the other parameters are constant.

**Figure 6.**
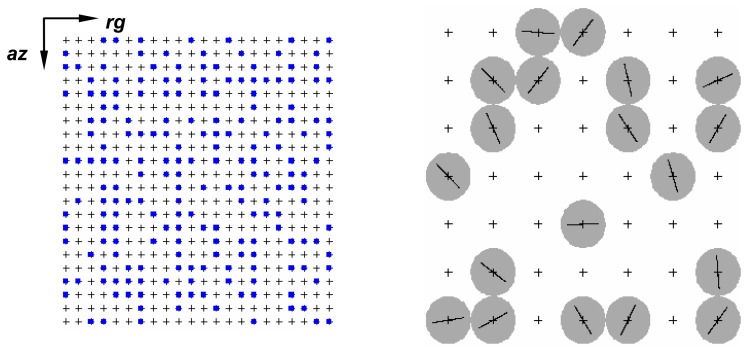
Rectangular grid with a few selected dipole positions and a zoomed area.

Image generation begins with the definition of a regular rectangular grid of coordinates over the scene to be imaged. The grid size is determined by the dipole size and by SAR operational parameters, such as spatial resolution, pixel spacing and the extension of the area to be imaged. The coordinates of the rectangular grid determine the possible positions that any structure can occupy. Mutual interaction among the dipoles can be avoided by the definition of a guard band around each one. In Figure 6 is illustrated a rectangular grid showing a few selected dipole positions (blue points) and a zooming part depicting the guard band (gray circle) and the dipole orientation. In the simulation process it is assumed that there is no dominant scatter in the image. In addition, according to [13] six elementary scatters are sufficient, in practice, to the components in-phase (*I*) and quadrature (*Q*) of received backscattered signal have Gaussian distribution with zero mean and equal standard deviation *σ*. Taking this statement into account, the simulation process used in this work guarantees at least fifteen elementary scatters within each resolution cell. The position and the local orientation of the dipoles, in the *xy*-plane, are uniformly distributed; their local orientation is distributed over the interval of 0° to 180°.

Considering that the multilayer structure is excited by (vertically and horizontally) polarized plane waves, the far electric field scattered by the structure is computed based on the electromagnetic model and the stationary phase method. In the spherical coordinate system, this field is given by (20), where it refers to the scattering element whose phase center is at the origin of the coordinate system. Each individual field scattered by a structure will contribute to the total return observed in each resolution cell. It is important to mention that due to the location of each scatter element phase center will appear an additional phase factor in its scattered field. This phase factor is given by exp{*i k_0_d_m_* cos*ζ_m_* }, where *d_m_* is the Euclidean distance to phase center of the *m*-th element and cos *ζ_m_* = cos *ξ_m_* sin*θ*cos*φ*+ sin *ξ*_m_ sin*θ*cos*φ*, with *ξ_m_* the angle measured counterclockwise from the *x*-axis to the distance line.

In order to form an image pixel the radar return is calculated by the vector summation of the individual fields weighted by a separable two-dimensional sinc(.) function (32). This function is introduced in the image generation process to represent the SAR spread point function, which takes into account the spatial correlation of the contiguous pixels in SAR image. The weight values are estimated based on a neighborhood of each resolution cell. The two-dimensional separable sinc(.) function is given by

(32) $$h(x,r)=h_{0}\sin c\left(\frac{\pi }{\delta_{a}}x\right)\sin c\left(\frac{\pi }{\delta_{r}}r\right),$$

where *x* and *r* represent, respectively, the terrain azimuth and range coordinates, *h*_0_ is a proportionality constant and the required spatial resolutions (in the azimuth and range directions) for the SAR image are expressed by *δ_a_* and *δ_r_*, respectively. Note that the *xy*-plane coincides with the azimuth-ground range plane.

### 3.1. Simulated Images

Multifrequency sets of single-look polarimetric SAR images have been generated in the L-, C- and X-bands, corresponding to 1.25, 5.3 and 9.6 GHz respectively. The acquisition geometry is particularized for a monostatic sensor flying at an altitude of 6,000 m (airborne platform altitude) and 35° grazing angle imaging a 290 m × 290 m area terrain. For this imaging geometry, the look angle between near- and far-range changes less than 1°, that is a small variation around of the look angle at the center swath width. The 3.0 m spatial resolution and 2.8 m pixel spacing were set in the range and the azimuth directions. In the simulation process the elementary scatterer was represented by a 50 mm × 1 mm electric dipole printed on the interface *z* = *d*_2_ (see the structure in Figure 2). A 9 × 9 pixels square window was used to determine the neighborhood for the estimates of sinc(.) function weights.

**Figure 7.**
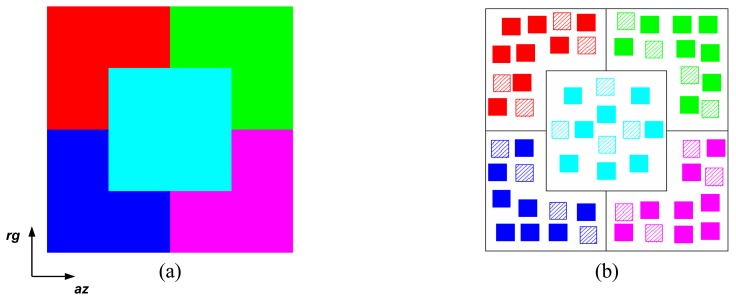
Phantom image and sample locations.

The simulated images are based on a phantom image (an idealized cartoon model), which contains five different regions. The phantom image is depicted in Figure 7a. The analysis that follows is based on a set of twelve 10 × 10 pixels square samples for each image region, as shown in Figure 7b. Each set is made up by eight training (the solid polygons) and four test (the hachured polygons) samples for the classification analysis performed in section 4.3.

Differentiation among the image regions is based on the local orientation of the dipoles and the electric characteristics of the layers. The dipole local orientation is relative to the azimuth-axis (*az*), and the coordinate system origin is located at the center of the image. The main characteristics of each region of the structure are summarized in Table 1, where TR stands for ‘totally random’. It is assumed that the magnetic permeability of all layers is *μ*_0_ and that the confined layers (layers 1 and 2 of the structure) have the same electric characteristics and thickness (*ℓ_i_*), equivalent to 1.1 L-band wavelengths. As observed from Table 1, the main difference among regions A, B and C is the local orientation, whereas the relative electric permittivity and the loss tangent of the confined layers distinguish regions C, D and E.

**Figure 8.**
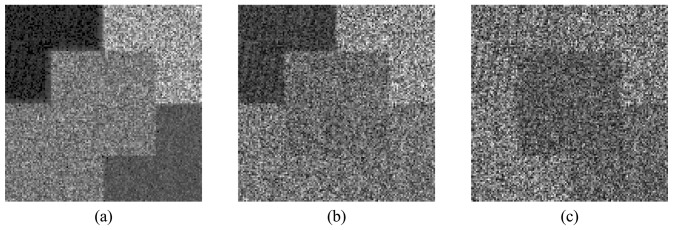
L-band amplitude simulated polarimetric SAR images: (a) HH, (b) HV and (c) VV channels.

**Figure 9.**
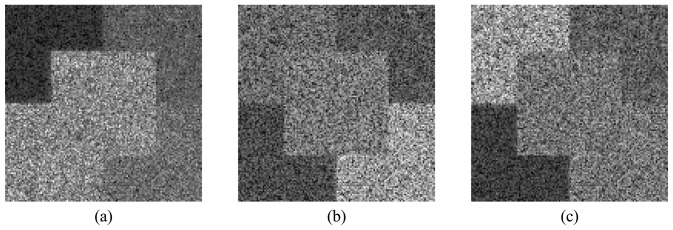
C-band amplitude simulated polarimetric SAR images: (a) HH, (b) HV and (c) VV channels.

**Figure 10.**
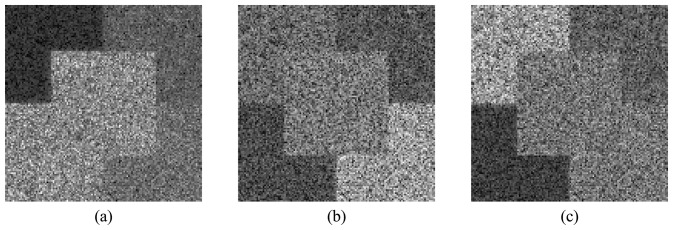
X-band amplitude simulated polarimetric SAR images: (a) HH, (b) HV and (c) VV channels.

Figures 8,9 and 10 show the HH, HV and VV simulated amplitude channels for the three bands. The VH channel is not considered, since it is the same as the HV one, due to the reciprocity assumption. It is important to mention that system (*r̂*,*θ̂*,*ϕ̂* of a standard spherical coordinate system was chosen to correspond to the *(k̂*,*v̂*,*ĥ*) coordinate system [14] in the simulation process.

**Figure 11.**
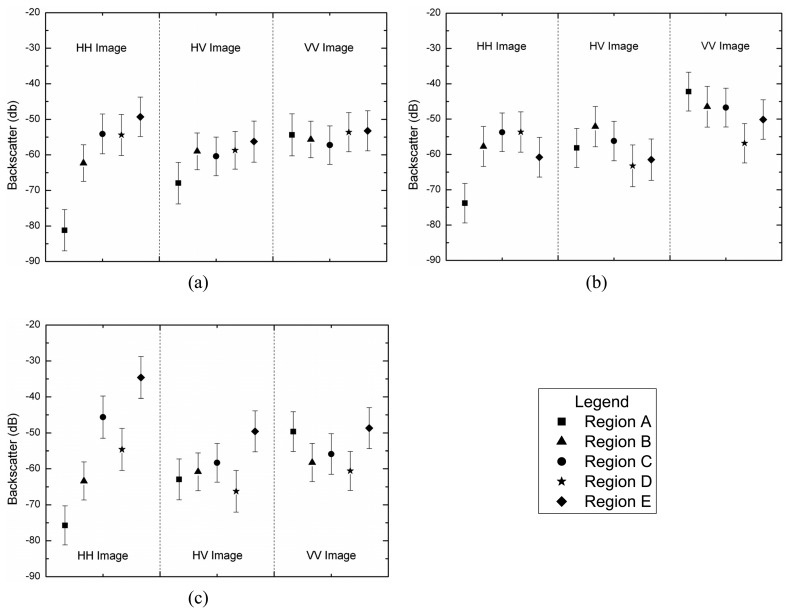
Mean backscatter values for each image region, per channel: (a) L-, (b) C- and (c) X-bands.

The first part of the SAR data analysis carried out is visual inspection (qualitative analysis). It shows that all images present a granular appearance typical of speckle noise. Mainly in the HV and VV channels, the boundary between some regions is unclear, due to the speckle noise effect. The mean backscatter plots, shown in Figure 11, where the error bars represent one standard deviation, reinforce the visual perception relative to the regions discrimination. From these graphics it can be seen that region A of the HH channel presents the lowest backscatter level in all bands, becoming the only region that can be clearly distinguishable from the others. In general, each region's backscatter depends on the frequency band.

In the L-band, regions C and D of the HH channel have similar backscatter mean values, off by about 0.28 dB, which makes extremely difficult the separation between these regions. The largest difference between the mean backscatter levels among regions B, C and D of the HV channel is about 1.73 dB, for which the visual distinction between regions is already problematic. For the VV channel, regions A, D and E, as well as regions B and C, present the same separability issue. This fact can be confirmed by visual inspection of the HV and the VV channels, where distinction between regions becomes a hard task, except for region A of the HV channel.

In the C-band, the ability to discriminate the image regions is an issue for regions C and D in the HH channel, for the pairs of regions A-C and D-E in the HV channel, and for regions B and C in the VV channel. The largest difference between the mean backscatter levels for these regions reaches 1.94 dB. For the HH channel in X-band, all regions are visually distinguishable since the boundaries between any two contiguous regions can be clearly identified. This statement is not true however for regions B and C of the HV channel, as well as for regions A and E and for regions B and C of the VV channel, where the largest difference between the mean backscatter levels reaches 2.5 dB. Consequently, it can be stated that, for any variation in the backscatter mean levels that is less than 2.5 dB, the two corresponding regions will not be visually distinguishable.

## 4. Image Analysis

The purpose of this section is to carry out a quantitative analysis in the simulated images aiming at the validation of the simulation methodology. The analysis will be performed through statistical tests and feature extraction from SAR amplitude and polarimetric data. An application employing the simulated data is also shown using two classification procedures.

### 4.1 Amplitude Data

Within the SAR image processing community, the multiplicative model is widely used to describe statistically the data [4], [15]. From this modeling, it is well known that the amplitude SAR data (linear detection) from homogeneous areas obey a square root of gamma distribution, once for constant terrain backscatter (no texture) the observed variation is due to speckle noise. The square root of gamma is a two-parameter family of continuous distributions, having a scale parameter *β* and a shape parameter *n*. This distribution can be written for every *x* > 0 by:

(33) $$f_{X}(x)=\frac{2n^{n}}{\beta^{n}\Gamma (n)}x^{2n-1}\exp\left\{-nx^{2}/\beta \right\},\;\beta >0,n\geq 1,$$

where parameter *n* stands for the equivalent number of looks (*ENL*). The *n* and *β* parameters are estimated using an interactive procedure [21] based on the first and the second order moments of each selected sample.

**Figure 12.**
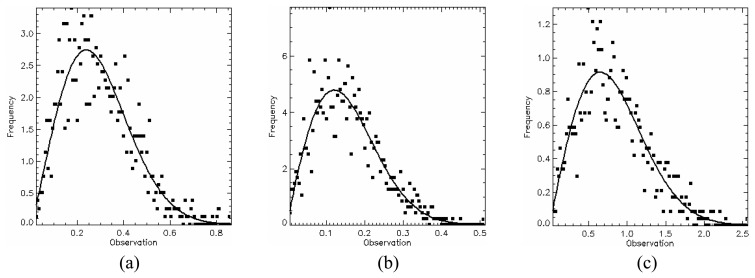
L-band fits: (a) HH - region B, (b) HV - region A and (c) VV - region E.

**Figure 13.**
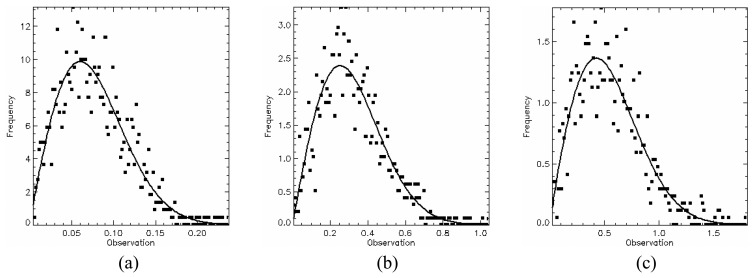
C-band fits: (a) HH - region A, (b) HV - region E and (c) VV - region D.

**Figure 14.**
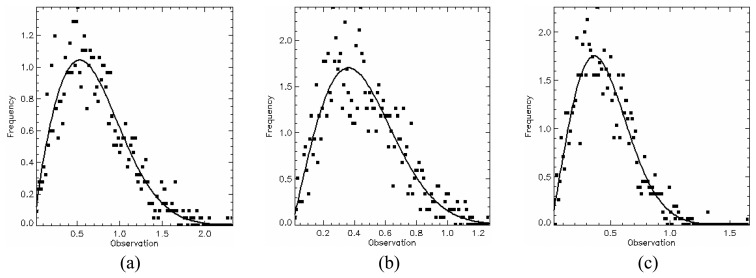
X-band fits: (a) HH - region D, (b) HV - region C and (c) VV - region B.

In order to test the hypothesis that the generated SAR data has a square root of gamma distribution, a *χ*^2^ goodness-of-fit test was performed for all channels using only one sample of each image region. This sample has size of 1200 pixels since it was formed by grouping the training and test sample sets. The test was applied to amplitude data and their resulting p-values are listed in Table 2. The lowest p-value (11.51%) occurs for HV channel in C-band. Therefore, there is no evidence to reject the hypothesis that all regions for any channel in the three bands are homogeneous areas and exhibit a square root of gamma distribution. The p-values of *χ*^2^ goodness-of-fit test for each channel in each band are shown in Table 2, where the largest p-values are typed in bold face. The data histogram (square symbols) and their corresponding fitted distribution (solid line) for these largest p-values are shown in Figures 12,13 and 14.

Another important quantity, commonly used within the SAR literature, is the equivalent number of looks, which can be estimated from the moments of the square root of gamma distribution. The estimated *ENL* was used as a quantitative measure for evaluating the one-look, using the individual samples of 100 pixels. The final estimates of *ENL* in each image region were computed as the average of those individual estimates, and are presented in Table 3. The estimated values are very close to one, as expected for a single look data.

Under the linear detection and for single look data the ratio of the standard deviation and the expected value (the *C_v_*) over homogeneous area is constant and equal to [(4-*π*)/*π*]^1/2^ = 0.5227. This value can be obtained from the moments of the Rayleigh distribution, which is a particular case of the square root of gamma when *ENL* is equal to one. As a consequence, another simple way of evaluating the simulated data is to check whether the mean value (*μ*) and the standard deviation (*σ*) of the data holds the linear relationship *σ*= 0.5227×*μ* within homogeneous area. This analysis was performed by applying a simple linear regression model [16] to the estimated means and standard deviations for all samples in each channel that were properly fitted by a square root of gamma distribution. The fitted linear regression model states that *Y* = *b*_0_+*b*_1_×*X*, where *b*_0_ and *b*_1_ are the estimated intercept and slope, respectively. In the case under analysis it is expected that the intercept should be zero and the slope should be equal to 0.5227.

**Figure 15.**
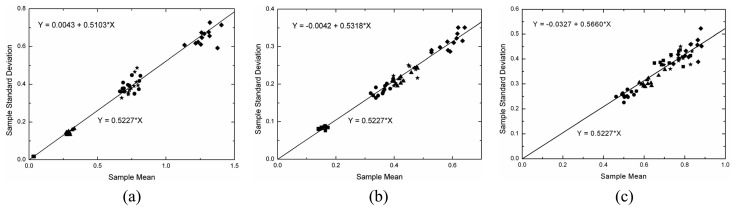
Linear fit to L-band sample data: (a) HH, (b) HV and (c) VV channels.

**Figure 16.**
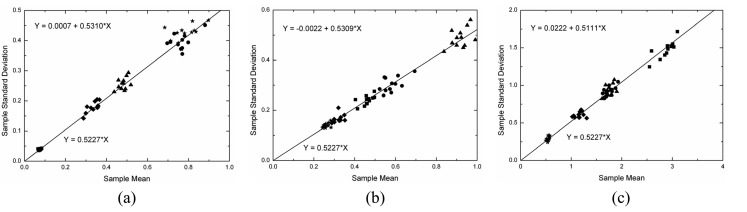
Linear fit to C-band sample data: (a) HH, (b) HV and (c) VV channels.

**Figure 17.**
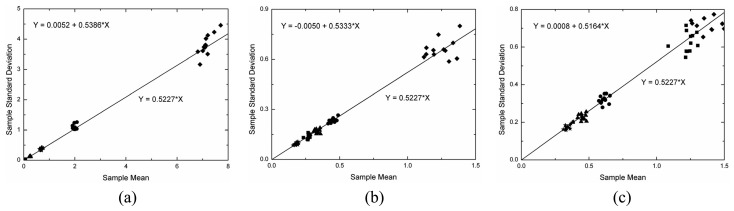
Linear fit to X-band sample data: (a) HH, (b) HV and (c) VV channels.

The adjusted linear model (*Y* = *b*_0_+*b*_1_×*X*), where *Y* represents the standard deviations and *X* the mean values, and the expected linear model (*Y* = 0.5227×*X*) are depicted on Figures 15 and 17, respectively for L-, C- and X-bands in each channel. In these figures the straight line is represented by the expected linear model and the symbols follow the same legend adopted in Figure 11, i.e., the square, the triangle, the circle, the star and the diamond represent the regions A, B, C, D, and E, respectively. The obtained straight line for each channel is not shown in these figures because it is very close to the expected one. By observing the Figures 15,16 and 17 it can be noted that the estimated values for the intercept (*b*_0_) are always around zero and, in general, the values of slope (*b*_1_) are over estimated, but around the 0.5227 value.

A Student's t-test for the intercept being equal to zero and the slope being equal to 0.5227 was performed. The number of samples used in each linear fit and their respective p-values for statistical analysis of the two parameters of the regression are shown in Table 4. From the number of samples it is possible to note that few samples (approximately 3%) were not fitted well. Analyzing the p-values is observed that only one value lower than 5% is encountered for the slope test in HH channel of X-band, that is, for this case there is no evidence to accept the hypothesis of the slope to be equal to 0.5227.

### 4.2 Polarimetric Data

A major problem in analyzing polarimetric SAR data arises from the complexity of the scattering mechanisms that give rise to features in the different polarization parameters. A lot of work has been done for modeling polarimetric radar backscatter for various types of targets. In [17], for example, the relationship between the HH-VV polarization phase difference (*PPD*) at P-band and some forestry parameters, such as diameter at breast height, stand age, basal area and truck biomass for a pine plantation was analyzed. Similarly Durden *et al.* [18] reported the *PPD* returns show that the ground/tree interaction is important at P-band. Moreover, in [19], is presented a technique for unsupervised classification of scattering behavior by selecting the dominant scattering mechanism, based on *PPD*, where each pixel is classified as either an odd number of reflection (small *PPD* values), even number of reflection (large *PPD* values) or diffuse scattering. A *PPD* of 0° means that the scattering mechanism is a single scattering, whereas a *PPD* of 180° suggests that the scattering mechanism be a double-bounce scattering.

**Figure 18.**
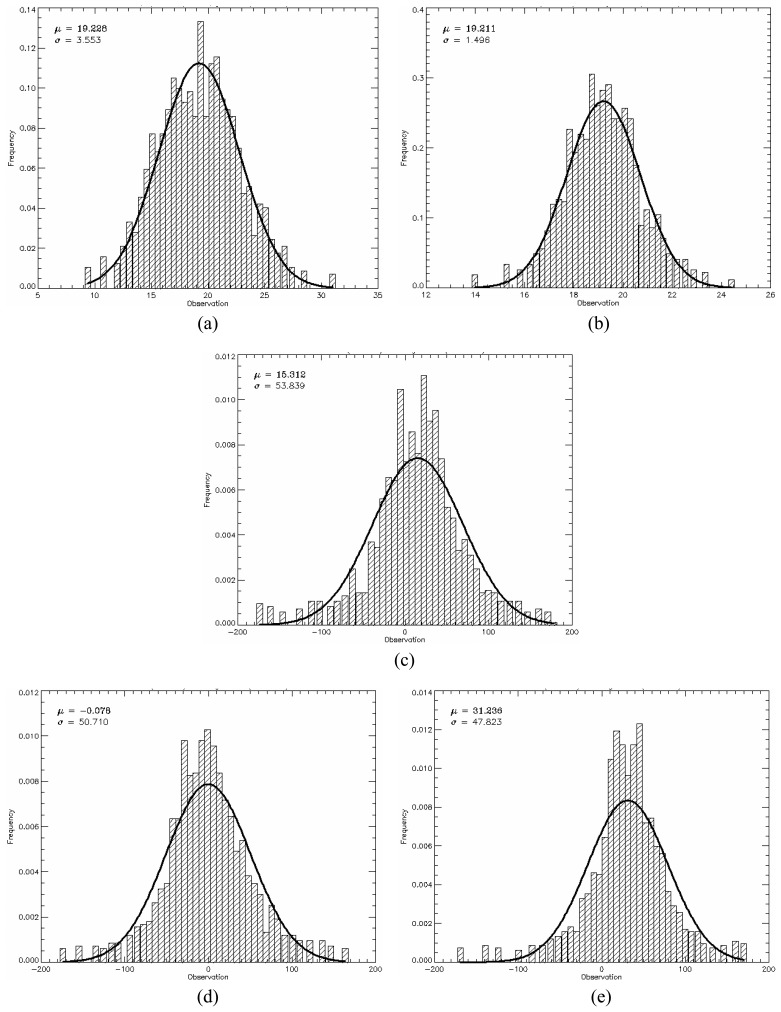
L-band *PPD*'s histogram for all regions in comparison to theoretical Gaussian distribution (solid line).

**Figure 19.**
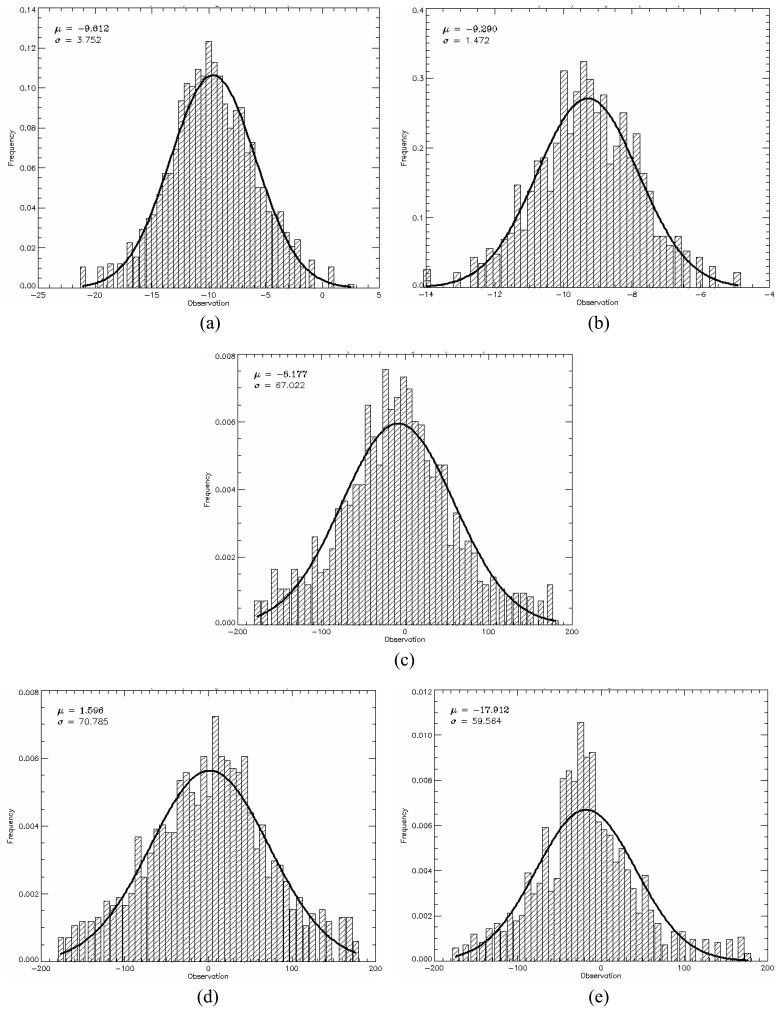
C-band *PPD*'s histogram for all regions in comparison to theoretical Gaussian distribution (solid line).

**Figure 20.**
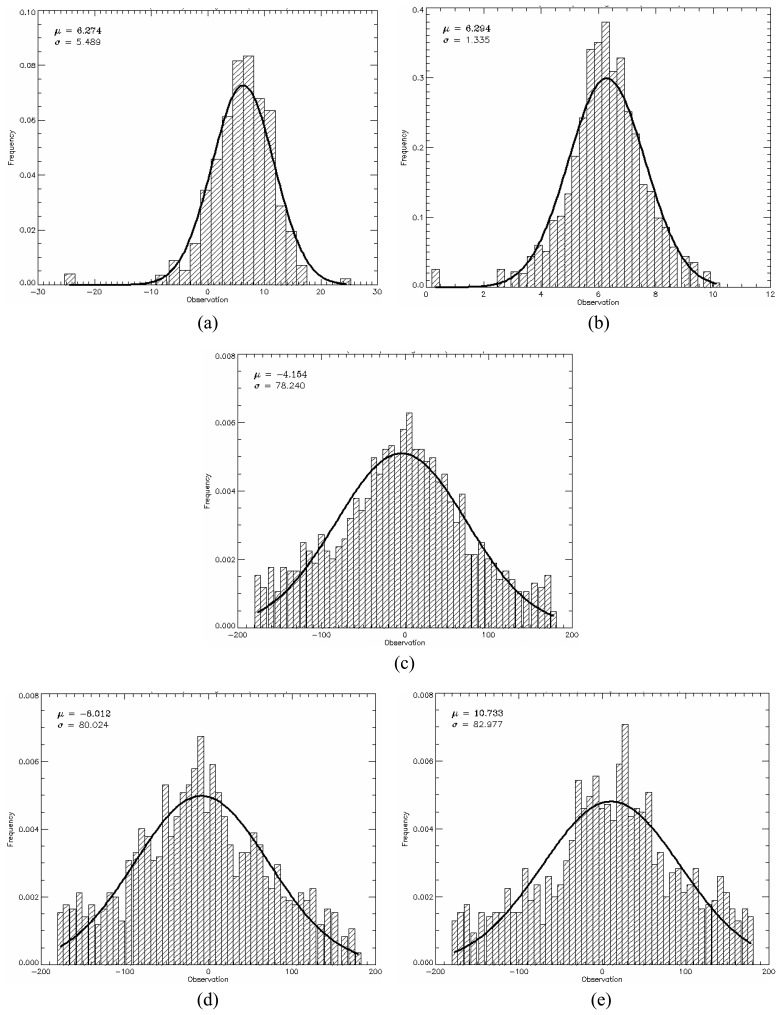
X-band *PPD*'s histogram for all regions in comparison to theoretical Gaussian distribution (solid line).

The *PPD*, given by (34), was used as quantitative measure to evaluate the generated images from the point of view of polarimetric data. The mean *PPD* values and its corresponding standard deviation (shown in Table 5) were computed for each image region in all bands, based on the twelve samples, as well as their histograms shown in Figures 18,19 and 20 for L-, C- and X-bands, respectively. In these figures the image regions are presented in alphabetic order.

(34) PPD=tan−1(−Im(Shh∗Svv)Re(Shh∗Svv))

The histograms of *PPD* attribute appear to be symmetric for all bands and all type of image region. It was also performed a Kolmogorov-Smirnov goodness-of-fit test verifying that *PPD* values can be derived from a Gaussian distribution. The p-values obtained for these fits are shown in Table 5, all being greater than 30.56%. In Figures 18,19 and 20 a comparison with theoretical Gaussian distribution plotted over the histograms is also illustrated.

The mean values of *PPD* attribute ranges from -17.91° to 31.24°, low values, which suggest that the major scattering mechanism is characterized by a single scattering (single bounce) for all image region. On the other hand, the standard deviations of *PPD* vary considerably with radar band and region type over the range 1.34° to 82.98°. The high values (consistently greater than 47.82°) for regions C, D, and E are indicators that these regions present structural heterogeneities. Due to the low values of standard deviations of the *PPD* the regions A and B can be seen as targets having uniform scattering properties (homogeneous structure).

The polarimetric images analysis follows by deriving some polarimetric features from the standard Cloude-Pottier eigenvalue/eigenvector target decomposition [20]. These features are deduced from the decomposition applied on the average coherency matrix. Under the reciprocity assumption framework and a monostatic measurement the coherency matrix can be expressed as a linear combination of the outer products of three eigenvectors. This means that the average coherency matrix can be decomposed into a sum of three independent scattering mechanisms, since each eigenvector corresponds to one scattering matrix.

The eigenvalue/eigenvector decomposition of the coherency matrix into elementary mechanisms (i.e. single, double and volume scattering) is employed in order to identify the global mean scattering mechanism. From the eigenvalue/eigenvector can be defined the ᾱ-angle, which ranges from 0° to 90° and is used to represent physical scattering mechanism. Furthermore, eigenvalues can be combined to form the anisotropy (*A*) and the entropy (*H*) parameters, scalar quantities ranging from 0 to 1; the former is a measure of the degree of randomness the scattering process and the latter is a complementary measure to *H*, related to secondary scattering mechanism. Low entropy (*H* ≈ 0) indicates a single scattering mechanism (isotropic scattering) while high entropy (*H* ≈ 1) indicates a totally random mixture of scattering mechanisms with equal probability and hence a depolarizing target.

From the eigenvalue analysis it was observed that for all image regions their coherency matrix has only one nonzero eigenvalue (coherency matrix with rank 1). It leads to a zero entropy, which complies with a deterministic scattering process (or pure target), characterizing a single scattering matrix equivalent descriptor. It means that the region does not depolarize the incident wave, and in this case the anisotropy is zero also.

**Figure 21.**
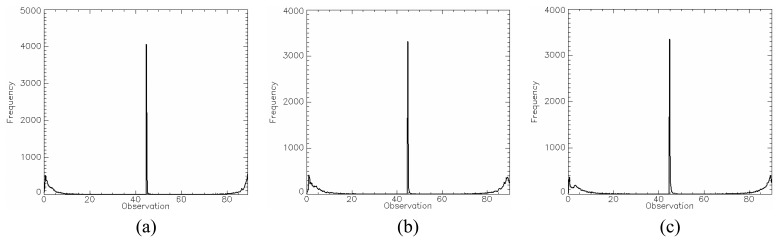
Alpha image histogram: (a) L-, (b) C- and (c) X-bands.

A pointwise estimation was employed to form an ᾱ image in all bands and their histograms are illustrated in Figure 21. This behavior is analogous in all regions, indicating that they have similar scattering mechanisms. Note that the greatest peak (maximum occurrence) is around 45°, suggesting that there exists a high percentage of volumetric scattering mechanism in the image regions and low contribution of the surface scattering (*ᾱ* = 0°) and the double bounce scattering (*ᾱ* = 90°). In addition, the similarity among the region leads to a low discriminatory capability based on ᾱ. The histograms reinforce the remark that each region has a deterministic scattering process, since as stated in [20], an ᾱ value equal to 45° characterizes a dipole scatter mechanism. This result is expected because all regions were created with the same dipole having only different local orientation angles.

### 4.3 Data Classification

Digital classification is one of the most extensively used tools in remote sensing applications. Using this tool the discriminatory capability of polarimetric generated images is quantitatively evaluated. The classification procedure used is based on the Iterated Conditional Modes (ICM) algorithm [21-23], that is a supervised procedure.

The ICM method is a contextual procedure that, in order to classify every pixel, uses both the observed value in the corresponding co-ordinate and the classification of the surrounding sites. In order to incorporate the context within a statistical framework, a Markovian model is used for the classes. This model is known in the literature as Potts model [24]. The ICM algorithm consists of the iterative improvement of the classification of the co-ordinate *s*, using the information of its return and the classes of its neighboring sites. This improvement is obtained by maximizing the a posteriori distribution of classes given the observation and the surrounding classes, which is given by:

(35) $$L(\xi )=f_{\xi }(z_{s})\exp(\beta \#\{t\in \partial_{s}:\xi_{t}=\xi \}),$$

where *f_ξ_*(*z_s_*) is the density associated to class *ξ*, which has radiometric value *z_s_* on co-ordinate *s*, *β* is a real parameter that quantifies the influence of the neighboring class and it is estimated iteratively and *∂_s_* is the set of neighboring co-ordinates around site *s*. The iterative technique stops according to the number of co-ordinates whose classification changes from one iteration to the next [21]. The expression of *L*(*ξ*) can be reduced to the Maximum Likelihood (ML) classifier and to Mode Filter, when *β* = 0 and *β*→∞, respectively. For more details of the algorithm, the reader is referred to [21, 22, 25].

In order to evaluate the discriminatory capability of the five image regions two classification approaches based on the ICM classifier were applied. The first one takes into account the bivariate distribution of the HH and VV intensities channels developed in [26, 27]. The distribution of the pair of intensities arises from the multivariate complex Wishart distribution [28], which models the covariance matrix of the multilook polarimetric data, since it was assumed that the speckle obeys a multivariate complex Gaussian law [29] and the terrain backscatter has a constant distribution (no texture). The second classification approach is based on two polarimetric attributes derived from the HH and VV backscattering coefficient. The former is called by polarization discrimination ratio (*PDR*) was proposed in [6] and the later is denoted here as polarimetric description square root (*PDS*) being proposed in [30]. Both attributes aim for the retrieval of soil moisture from SAR data, and are, respectively, given by

(36) $$\text{PDR}=\frac{\sigma_{\text{vv}}^{0}-\sigma_{\text{hh}}^{0}}{\sigma_{\text{vv}}^{0}+\sigma_{\text{hh}}^{0}},$$

(37) $$\text{PDS}=\sqrt{\sigma_{\text{vv}}^{0}\sigma_{\text{hh}}^{0}},$$

where $\sigma_{\text{pq}}^{0}$ represents the backscattering coefficient for *pq* polarization. It is important to mention that, in this second classification procedure, it was assumed a bivariate Gaussian distribution to model the joint density of the *PDR* and *PDS* attributes.

**Figure 22.**
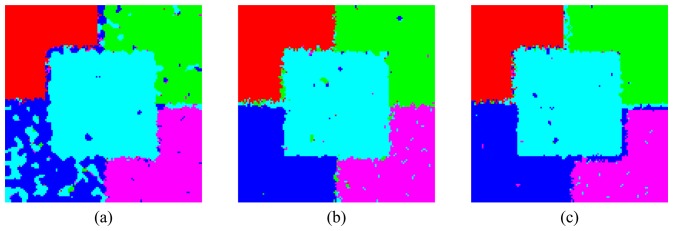
Classified images using bivariate HH-VV distribution: (a) L-, (b) C- and (c) X-bands.

**Figure 23.**
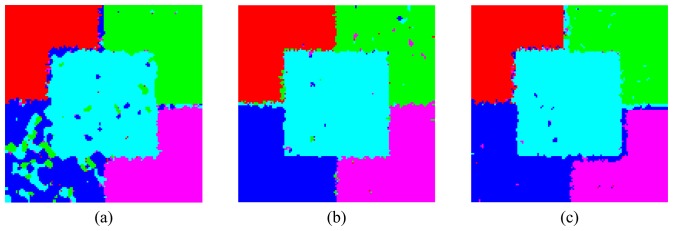
Classified images using the *PDR* and *PDS* features: (a) L-, (b) C- and (c) X-bands.

The results of the classification are shown in Figures 22 and 23 for both approaches. The performances of the ICM classifications are quantified through the kappa coefficient of agreement estimative (*κ̂*) [31, 32] which includes the estimation of the a priori probabilities of classes based on the number of training or test examples. The estimates of *κ̂* and its corresponding variance ( $\sigma_{\hat{\kappa }}^{2}$) were computed for all classifications, based on confusion matrices being presented in Tables 6,7,8,9,10 and 11.

The classification results can be considered excellent in both approaches and for all bands, since all regions could be well distinguished from the others with little confusion among them. The results show the sensibility of the HH and VV channels and the attributes *PDR* and *PDS* to variations in the electric characteristic of the regions as well as variations in the elementary scatter orientation. This fact leads to further theoretical studies, using the simulated polarimetric images, aiming the extraction of geophysical parameters. In general, L-band presents the worst results of classification followed by C- and X-bands. In L-band the greatest confusion is found between C and D regions, followed by the confusion between C and E regions. On the other hand the confusion between B and C regions is the largest one when using C-and X-bands. These results suggest that the L-band might not be so adequate as C- and X-bands to conduct studies of the sensitive of electric characteristic of a target to microwave frequencies.

A two-sided statistical z-test was performed to evaluate the equality between all pair of *κ̂* values. The tests of equality of two pairs of *κ̂*, at a significance level of 95%, demonstrated that all classifications obtained using C- and X-bands can be considered statistically equal. In this way, for the used parameter characteristic of each region and classification approaches, C- and X-bands presented the same performance. In contrast, in L-band the bivariate HH-VV classification is statistically equal only to the classification obtained by *PDR*-*PDS* attributes for L-band, showing that the information gathered by HH and VV channels is not significantly changed when these channels are combined in the *PDR* and *PDS* attributes for classification purposes.

## 5. Conclusions

This paper presented an electromagnetic way to simulated polarimetric SAR images, starting from Maxwell's equations. Images were simulated with five different regions, and their electromagnetic characteristics were used to distinguish them. The generated images were evaluated according to several measurements commonly employed in SAR data analysis. Firstly, the evaluation analysis consisted of statistical tests to amplitude data, showing that the data are adequately fitted by a square root of gamma distribution, which is the characteristic distribution of the multilook amplitude SAR data. Secondly, the equivalent number of looks was estimated, proving that the simulated data have only one look as simulated. It was checked by using a simple regression linear model whether the mean value (*μ*) and the standard deviation (*σ*) of the data exhibit the linear relationship *σ*= 0.5227×*μ* within homogeneous areas. From regression linear analysis it can be concluded that this relationship holds for all simulated images. It was also analyzed the polarimetric content of the data based on the HH-VV polarization phase difference (*PPD*) and the standard Cloude-Pottier eigenvalue/eigenvector target decomposition. The polarimetric evaluation revealed that the simulated data can be used as polarimetric SAR data, since the results are in accordance with those found in the literature. Finally, it was assessed the discriminatory capability of the image regions by applying two classification approaches based on the ICM classifier. The classification results showed that C- and X-bands have greater discriminatory power than L-band, for the parameters used to describe each region. Consequently, from the evaluation results can be affirmed that the simulation process is adequate and the simulated polarimetric data are in good agreement with those produced by a SAR sensor. Furthermore this simulation process can used to improve the understanding of SAR data properties in different situation, images can be generated to do theoretical as well as practical studies in several SAR subjects. For example, studies of the sensitivity of the relative electric permittivity and loss tangent of a target to certain microwave frequency can be carried out, which is an important topic in the retrieval of soil moisture content from SAR data.

## Figures and Tables

**Table 1.** Region characteristics.

**Region**	**Color**	***ε*_r_**	**tan*δ*_r_**	***ε_r_*_g_**	**tan*δ*_g_**	**Dipole Orientation**
**A**	Red	2.33	1.2×10^-4^	5.0	2.0×10^-1^	10°
**B**	Magenta	2.33	1.2×10^-4^	5.0	2.0×10^-1^	30°
**C**	Cyan	2.33	1.2×10^-4^	5.0	2.0×10^-1^	TR
**D**	Blue	4.00	1.2×10^-1^	8.0	2.0×10^+1^	TR
**E**	Green	2.33	1.2×10^-4^	8.0	2.0×10^+1^	TR

**Table 2.** P-values for the *χ*^2^ goodness-of-fit.

**Region**	**p-value (%)**								

	**HH**			**HV**			**VV**		
	**L**	**C**	**X**	**L**	**C**	**X**	**L**	**C**	**X**
**A**	22.59	**92.44**	73.35	**79.30**	35.35	67.36	48.03	48.53	84.72
**B**	**79.46**	72.05	90.44	46.18 2	68.9	59.72	51.89	41.01	**95.50**
**C**	31.52	87.14	79.49	38.00	24.35	**69.15**	36.07	46.24	78.61
**D**	72.66	72.78	**98.72**	77.65	11.51	68.92	52.18	**68.92**	33.27
**E**	68.24	52.09	46.94	41.74	**84.77**	57.41	**82.88**	48.68	44.35

**Table 3.** Estimated Equivalent Number of Looks (*ENL*).

		L			C			X		

Average ENL	Region	HH	HV	VV	HH	HV	VV	HH	HV	VV
**Per Samples**	**A**	0.984	0.972	0.958	1.003	1.022	1.037	1.047	1.068	1.084
	**B**	1.118	1.117	1.113	0.985	0.986	0.988	1.083	1.087	1.093
	**C**	0.981	1.041	1.113	1.049	1.028	0.999	0.917	1.030	1.005
	**D**	1.020	1.050	1.017	0.944	0.976	0.979	0.934	1.019	1.040
	**E**	1.072	0.998	1.011	0.978	1.038	1.008	0.985	0.994	1.034

**Per Region**		1.035	1.035	1.042	0.992	1.010	1.002	0.993	1.040	1.051

**Per Band**			1.038			1.001			1.028	

**Table 4.** Statistic for the regression linear fit.

**Band**	**Channel**	***n***	**p-value (%)**	

			***b*_0_**	***b*_1_**
**L**	**HH**	59	53.02	16.26
	**HV**	56	36.99	39.37
	**VV**	59	8.44	10.64
**C**	**HH**	57	90.74	44.36
	**HV**	59	74.73	49.60
	**VV**	59	16.45	20.60
**X**	**HH**	58	80.63	0.87
	**HV**	60	42.95	29.03
	**VV**	58	94.03	59.67

**Table 5.** *PPD* mean and standard deviation and p-value for the goodness-of-fit test.

**Region**	**L-band**	**p-value (%)**	**C-band**	**p-value (%)**	**X-band**	**p-value (%)**
**A**	19.228 (3.553)	99.63	-9.612 (3.752)	99.79	6.274 (5.489)	97.05
**B**	19.211 (1.496)	99.67	-9.290 (1.472)	99.99	6.294 (1.335)	97.27
**C**	15.312 (53.839)	54.24	-8.177 (67.022)	90.97	-4.154 (78.240)	99.83
**D**	-0.078 (50.710)	63.92	1.596 (70.785)	99.64	-8.012 (80.024)	98.94
**E**	31.236 (47.823)	30.56	-17.912 (59.564)	33.58	10.733 (82.977)	99.08

**Table 6.** Confusion matrix (# of pixels) using the bivariate HH-VV distribution (L-Band).

**Classification**	**Reference Data**					

	**A**	**B**	**C**	**D**	**E**	**Total**
**A**	**400**	0	0	2	0	402
**B**	0	**400**	0	1	0	401
**C**	0	0	**398**	61	14	473
**D**	0	0	2	**330**	6	338
**E**	0	0	0	6	**380**	386

**Total**	400	400	400	400	400	

*κ̂* = 0.943

$\sigma_{\hat{\kappa }}^{2}$ = 3.419 × 10^−5^

**Table 7.** Confusion matrix (# of pixels) using the bivariate HH-VV distribution (C-Band).

**Classification**	**Reference Data**					

	**A**	**B**	**C**	**D**	**E**	**Total**
**A**	**400**	0	1	0	0	401
**B**	0	**395**	1	0	0	396
**C**	0	5	**392**	0	0	397
**D**	0	0	6	**400**	0	406
**E**	0	0	0	0	**400**	400

**Total**	400	400	400	400	400	

*κ̂* = 0.992

$\sigma_{\hat{\kappa }}^{2}$ =5045 × 10^−6^

**Table 8.** Confusion matrix (# of pixels) using the bivariate HH-VV distribution (X-Band).

**Classification**	**Reference Data**					

	**A**	**B**	**C**	**D**	**E**	**Total**
**A**	**400**	0	0	0	0	400
**B**	0	**396**	0	0	0	396
**C**	0	4	**398**	1	0	403
**D**	0	0	2	**399**	0	401
**E**	0	0	0	0	**400**	400

**Total**	400	400	400	400	400	

*κ̂* = 0.996

$\sigma_{\hat{\kappa }}^{2}$ =2.725 × 10^−6^

**Table 9.** Confusion matrix (# of pixels) using the Gaussian bivariate distribution for *PDR*-*PDS* (L-band).

**Classification**	**Reference Data**					

	**A**	**B**	**C**	**D**	**E**	**Total**
**A**	**400**	0	0	3	0	403
**B**	0	**400**	1	0	0	401
**C**	0	0	**375**	45	0	420
**D**	0	0	17	**331**	1	349
**E**	0	0	7	21	**399**	427

**Total**	400	400	400	400	400	

*κ̂* = 0.941

$\sigma_{\hat{\kappa }}^{2}$ =3.528 × 10^−5^

**Table 10.** Confusion matrix (# of pixels) using the Gaussian bivariate distribution for *PDR*-*PDS* (C-band).

**Classification**	**Reference Data**					

	**A**	**B**	**C**	**D**	**E**	**Total**
**A**	**400**	1	0	0	1	402
**B**	0	**398**	0	0	0	398
**C**	0	0	**400**	0	2	402
**D**	0	0	0	**400**	0	400
**E**	0	1	0	0	**397**	398

**Total**	400	400	400	400	400	

*κ̂* = 0.997

$\sigma_{\hat{\kappa }}^{2}$ =1.948 × 10^−6^

**Table 11.** Confusion matrix (# of pixels) using the Gaussian bivariate distribution for *PDR*-*PDS* (X-band).

**Classification**	**Reference Data**					

	**A**	**B**	**C**	**D**	**E**	**Total**
**A**	**400**	1	0	0	0	401
**B**	0	**397**	0	0	0	397
**C**	0	0	**399**	0	2	401
**D**	0	2	1	**400**	0	403
**E**	0	0	0	0	**398**	398

**Total**	400	400	400	400	400	

*κ̂* = 0.996

$\sigma_{\hat{\kappa }}^{2}$ = 2.337 × 10^−6^
